# Packages of Care for Mental, Neurological, and Substance Use Disorders in Low- and Middle-Income Countries: *PLoS Medicine* Series

**DOI:** 10.1371/journal.pmed.1000160

**Published:** 2009-10-06

**Authors:** Vikram Patel, Graham Thornicroft

**Affiliations:** 1 London School of Hygiene and Tropical Medicine, Department of Epidemiology and Population Health, London, United Kingdom; 2 Institute of Psychiatry, King's College London, London, United Kingdom

## Abstract

In a Perspective article, Vikram Patel and Graham Thornicroft introduce a new series in *PLoS Medicine* on mental health disorders in low- and middle-income countries that reviews the evidence for packages of care for ADHD, alcohol misuse disorders, dementia, depression, epilepsy, and schizophrenia.

**Linked Neglected Diseases Series**

This Perspective introduces a new series in *PLoS Medicine* on mental health disorders in low- and middle-income countries that reviews the evidence for packages of care for ADHD, alcohol misuse disorders, dementia, depression, epilepsy, and schizophrenia.

The world's poorer and less resourced countries face a significant burden of mental, neurological, and substance use (MNS) disorders. This burden will continue to grow as the epidemiological transition—the process by which low and middle countries see a rise in noncommunicable diseases—gathers pace [1].

Recent reports on the care of persons living with MNS disorders had two stark findings. First, there is enormous inequity in the distribution of specialist human resources, both within and between countries [2]. One country alone (the US) enjoys more psychiatrists than the world's most populous countries (India and China) and an entire continent (Africa) put together. Second, due to a combination of factors including scarce resources, there is an astonishingly large treatment gap for people with MNS disorders. Worldwide at least two-thirds of all persons with mental illnesses, for example, go untreated, and in low resource countries this figures exceeds 90% [3],[4].

These findings sit alongside a compelling evidence base indicating the strong social determinants of MNS disorders and the vicious cycle of deprivation and mental ill-health [5]. Flagrant abuses of human rights of people with MNS disorders have been documented at every level of the health system including, most disturbingly, in the very institutions that are supposed to cater to their needs [6]. In response to this global health scandal, the recently launched Movement for Global Mental Health (www.globalmentalhealth.org) has called for the immediate scaling up of services for people with MNS disorders to close the treatment gap, on the basis of principles of evidence and human rights.

Which treatments should be scaled up and how should these be delivered in settings where specialists are scarce—a common situation for the majority of the global population? Starting this week, with the article by Chowdhary and colleagues on depression [7], *PLoS Medicine* publishes a series of evidence-based reviews on packages of care for six MNS disorders. We were invited to be Guest Editors for this series. Each review follows a set format: the focus is on both the evidence of efficacy of specific treatments, as well as how these should be delivered. Indeed the routine delivery or implementation of such interventions is of vital importance. Therefore the series is concerned not only with questions about the treatments themselves (e.g., who should provide these, and in what settings they be provided), but also with improving access to these treatments to achieve optimal long-term clinical and social outcomes.

The six disorders are attention-deficit/hyperactivity disorder (ADHD), epilepsy, depression, schizophrenia, alcohol use disorders, and dementia. These disorders comprise the leading MNS causes of disease burden across the life course. This *PLoS Medicine* series is intended to be entirely complementary to the new World Health Organisation (WHO) Mental Health Gap (mhGAP) initiative [8], which will soon produce recommendations on the use of specific treatments in primary and community health care settings in low- and middle-income countries (http://www.who.int/mental_health/mhGAP/en/).

Although the specific treatments differ between disorders, there are also many shared themes related to the delivery of these treatments. Detection and diagnosis of more common disorders (such as depression or alcohol use disorder) may be reliably carried out using brief screening questionnaires. Less common disorders (such as schizophrenia and dementia) may need a two-stage case-finding procedure with probable cases being identified through community case-finding strategies followed by a diagnostic interview by an appropriately trained health worker. A combined package of pharmacological and psychosocial treatments are efficacious for the treatment of MNS disorders; however, not all persons need all treatments. The proposed packages recommend stepped-care models where treatments are tailored to the needs of each individual. People with almost all of these disorders need continuing care and help to maintain regular use of medication for extended periods to achieve optimal outcomes. In these respects, MNS disorders are similar to other chronic conditions, such as diabetes and cardiovascular disease [9].

Nonspecialist health workers can deliver, safely and effectively, treatments for MNS disorders within a functioning primary health care system [10]. However, collaborative care models, in which specialists play diverse roles of capacity building, consultation, supervision, quality assurance, and providing referral pathways greatly enhance the effectiveness and sustainability of such nonspecialist health worker-led care programs [11].

These shared characteristics suggest that the packages of care for MNS disorders can be integrated at three levels: (1) packages catering to individual MNS disorders can be integrated with each other; (2) such packages can be integrated with the treatment of other chronic conditions; and (3) the packages can also be integrated within the primary health care system by strengthening its capacity for management of chronic conditions [9],[12].

However, it is also clear that simply making care available is not sufficient to close the treatment gap; access will continue to be hampered by stigma and discrimination [6]. Building mental health literacy and implementing strategies for combating stigma and discrimination for the whole population are critically important.

It is also clear that, as with all chronic conditions, most of the evidence is derived from high-income countries. However, it is heartening to note that where attempts to replicate evidence on effectiveness have been attempted in low and middle income countries, the findings are consonant with those from high income countries, suggesting that such evidence may be generalizable across cultures. While the packages of care published in *PLoS Medicine* can provide clear guidelines to scaling up services for people with MNS disorders, there is a need to generate appropriate evidence to guide the delivery of such effective treatments and their scaling up within routine health care delivery settings. Research on culturally appropriate and acceptable treatments, delivery and scale-up is the now the most important research priority for global mental health [13].

We believe that the most innovative aspect of this series is its focus on the critically important element of “delivery.” To the best of our knowledge, the series is the first attempt to collect comprehensive reviews of six leading, and mostly neglected, MNS disorders in an open access venue that allows immediate and full access to everyone including those living and working in low and middle income countries. We hope this series serves as a valuable resource for health professionals, policy makers, and health workers working to improve the care and treatment of those struggling with MNS disorders in settings where specialist resources are scarce and where treatment gaps are large.

## Abbreviations

MNS: mental, neurological, and substance use
